# Gamification-based intervention for enhancing team effectiveness and coping flexibility: Randomized controlled trial

**DOI:** 10.3389/fpsyt.2022.941252

**Published:** 2022-07-25

**Authors:** Cecilia Cheng, Chor-lam Chau

**Affiliations:** Social and Health Psychology Laboratory, Department of Psychology, The University of Hong Kong, Pokfulam, Hong Kong SAR, China

**Keywords:** coping, stress, intervention, serious game, mental health, psychological well-being, group cohesion, team building

## Abstract

This study aimed to evaluate a newly developed gamification-based intervention of serious play training (SPT). A randomized controlled trial was conducted to assess the efficacy of the new intervention program in comparison with a widely adopted cognitive-behavioral training (CBT) program. Real-life work teams were recruited to enhance the ecological validity of outcome evaluation. The participants comprised 250 Chinese working adults (68% men; median age = 25 years, range: 18–40) who took part voluntarily. They were randomly assigned to the SPT, CBT, and waitlist conditions. For outcome evaluation, team effectiveness was the primary outcome, whereas coping flexibility was the secondary outcome. For explanation of outcome changes, group cohesion and discriminative thinking were tested as the hypothesized learning mechanisms. The results revealed that the SPT group alone reported greater team effectiveness over time, with an increase in group cohesion found to explain the improvement. Both the SPT and CBT groups reported greater coping flexibility over time, with discriminative thinking found to account for the beneficial changes. These findings provide initial evidence indicating the efficacy of utilizing the gamification approach in corporate training for team-building and personal coping.

## Introduction

The rapidly evolving nature of the work environment and contemporary organizational adaptations are intrinsically entwined with the multiple challenges of daily life faced by many employees today (1, 2). The 2008 global economic crisis and the ongoing COVID-19 pandemic have imposed immense amounts of stress on employees amid widespread concerns about job insecurity and layoffs (3, 4). A recent meta-analysis indicates that coping flexibility is the cornerstone of psychological adjustment to stressful life changes, as demonstrated by the positive associations between this coping skill and multiple mental health indicators such as subjective well-being and quality of life (5).

To tackle the issues arising from the ever-changing work and economic environments, many organizations provide their employees with training to improve their coping skills, with many corporate training workshops emphasizing the importance of flexible coping in mitigating work stress. Cognitive-behavioral training (CBT) is currently one of the most common approaches to stress management intervention (6, 7). Several review studies have documented the efficacy of workshops adopting cognitive-behavioral approaches in strengthening discriminative thinking skills that equip individuals to deploy flexible strategies for coping with an array of stressors, thus mitigating the risks of developing mental health problems such as psychological distress and psychosomatic symptoms (8, 9).

Systematic reviews also indicate that CBT generally focuses on the person, and is highly prescriptive, non-interactive, product-oriented, skill-based, and instructor-centered (10). Participants taking part in CBT acquire an array of cognitive-behavioral skills (e.g., problem-solving, relaxation) that help them to expand their personal resources and mitigate psychological distress experienced during stressful encounters (6, 11). However, both the socio-cultural constructivist theory of learning (12) and cognitive flexibility theory (13) postulate that knowledge constitutes both the derivation of a learner’s interpretations of his or her personal experiences and the process of meaning-making through active interactions with others. Accordingly, learners tend to actively “construct” their knowledge, with such construction consolidating their cognitive flexibility and problem-solving skills (14, 15). More broadly, most person-oriented CBT workshops fail to implement intervention strategies that promote group cohesion, which is highly valued in work settings in many collectivistic societies (16).

To address these important but unexplored issues, an alternative approach—serious play training (SPT)—was adopted in the present study. Serious play is a novel intervention technique that refers to the use of games to educate, train, and inform workshop participants; and serious games have been shown to be successful as a learning method for building skills to tackle real-life complex issues or tasks, such as change management (17). It could therefore be expected that serious games would play an important role within corporate training. The efficacy of this newly developed workshop was evaluated by comparing it with the widely adopted CBT approach in a Chinese setting.

SPT differs from CBT in two major ways. First, SPT was developed according to the principles of gamification, which refers to the utilization of game design elements in daily life contexts to enhance the motivation and engagement of workshop participants (18, 19). The gamification approach has been found to incentivize active engagement in the given intervention, as participants are immersed in the training process and find it to be enjoyable and intrinsically rewarding (20, 21). It has also been found effective in reinforcing collective problem-solving skills and promoting active learning (18) as well as improving health and bolstering mental wellness (22, 23).

Second, a theoretical approach underpinning the SPT intervention design is collectivism-oriented human resource management (24), whereas CBT programs are “imported” from the West with different extents of cultural adaptations. For SPT, the cultural management strategy highlights the collective generation of creative solutions and ideas through team-based structures, which aligns with the socio-cultural values (e.g., collectivism) and institutional context of many Chinese organizations. Implementing the principles of a culturally relevant strategy has been found to promote team reflexivity in Chinese teams, which in turn bolsters their innovation and job performance (25).

Applying the principles of collectivism-oriented human resource management, SPT’s gamification design and practices focus on (a) treating employee training and development as team endeavors; (b) foregrounding teamwork and group cohesion rather than individual performance; and (c) rewarding teams of individuals (24). SPT thus involves a range of group-based activities aimed at facilitating social engagement among participants, with such engagement bolstering effective communication, collaboration, and relational quality among team members (18). Adopting the gamification approach in a work setting, Luu and Narayan (26) reported stronger communication skills to be associated with higher degrees of both individual and group task satisfaction. Moreover, another gamification-based intervention fosters a more collaborative learning environment and more favorable evaluations among team members (27). Taken together, these findings suggest that the proposed SPT is likely to provide workshop participants with ample opportunities for intensive collaboration, thereby consolidating teamwork as the basis for effective job performance (25, 28).

The structure and modules of both the proposed SPT and the existing CBT are summarized in Table 1. As shown in this table, SPT aims at mitigating stressors specifically related to the work setting through gaming activities that facilitate collective decision-making and social resource accrual. Both of these team-based activities have been found to increase group cohesion among team members, which in turn improve team effectiveness and reduce work stress (29, 30).

**TABLE 1:** Descriptions of stress management modules of serious play training and cognitive-behavioral training programs.

Serious play training		Cognitive-behavioral training	
Aim of module	Content	Aim of module	Content
1. Orientation and understand mechanisms of stress	Psychoeducation: nature and mechanisms of stress, transactional stress model, and links between stressors and stress reactions	1. Orientation and understand mechanisms of stress	Psychoeducation: nature and mechanisms of stress, transactional stress model, and links between stressors and stress reactions
2. Develop cognitive flexibility skills	Identification of personal signs of stress and stress triggers, reflection of stressful experience, development of discriminative thinking skills to distinguish among the nature and demands of diverse stressors, development of cognitive restructuring skills to challenge automatic irrational thoughts and replace them with more realistic flexible ones	2. Develop cognitive flexibility skills	Identification of personal signs of stress and stress triggers, reflection of stressful experience, development of discriminative thinking skills to distinguish among the nature and demands of diverse stressors, development of cognitive restructuring skills to challenge automatic irrational thoughts and replace them with more realistic flexible ones
3. Develop response flexibility skills	Identification of personal coping style and its limitations, reflection of coping experience, importance of expanding coping repertoire, development of discriminative thinking skills to recognize differential coping effectiveness across stressful situations, acquisition of good-fit principle for effective strategy deployment	3. Develop response flexibility skills	Identification of personal coping style and its limitations, reflection of coping experience, importance of expanding coping repertoire, development of discriminative thinking skills to recognize differential coping effectiveness across stressful situations, acquisition of good-fit principle for effective strategy deployment
4. Develop collective problem-solving skills	Team building process and group dynamics: team knowledge formation and evolution, strategic planning and group support tools, creation of a diverse and inclusive work culture, conflict management, the art of giving constructive feedback, group reflection, outcome review	4. Develop problem-solving skills	Systematic problem-solving and solution-oriented coping activities: problems finding, problems shaping, listing of possible solutions, making choices and back-up plans, action execution, progress monitoring and reflection, outcome review
5. Manage stress together and accrue social resources	Role of a supportive social environment, importance of expanding social network and social capital, communication skill training for identifying and activating coping resources	5. Practice behavioral activation and relaxation skills	Behavioral activation skills to increase pleasant and reinforcing daily activities, practice of mindfulness and breathing techniques for relaxation
6. Review and devise plans for action and maintenance	Summary of intervention components, review of team and personal performance, importance of skill integration and transfer to daily life, and creation of plans for daily action and maintenance	6. Review and devise plans for action and maintenance	Summary of intervention components, review of personal performance, importance of skill integration and transfer to daily life, and creation of plans for daily action and maintenance

Despite the aforementioned differences in their program structure and focus, SPT and CBT were both designed to strengthen discriminative thinking, an essential skill for flexible adjustment to stressful life changes (31). Discriminative thinking refers to an individual’s ability to recognize a unique set of situational features characterizing a specific stressful event (i.e., cognitive flexibility); that is, the ability to determine, for example, whether the outcome of a given stressor is amenable to change through his or her own effort or whether the stressor will exert an undesirable impact on his or her long-term life goals (32, 33). After recognizing the unique features of a variety of stressors, discriminative thinking further enables a person to discern and differentiate among the demands of multiple stressors (i.e., response flexibility), resulting in the deployment of appropriate strategies that meet the specific situational demands (31).

Through training in discriminative thinking in both SPT and CBT, workshop participants are temporarily drawn away from the conscious processing of their work, endowing them with an opportunity to “incubate,” to allow the unconscious processing of work to take place (34). Unconscious work and task switching during the incubation stage is conducive to creative problem-solving and reduced mental fixation (35, 36), the latter of which is key to promoting cognitive and response flexibility (37). To evaluate the hypothesized efficacy of both SPT and CBT, the following hypotheses were tested.

*Hypothesis 1*: Participants who took part in SPT (vs. CBT and no skill training) will have higher levels of team effectiveness over time.

*Hypothesis 2*: The positive association between SPT (vs. CBT and no skill training) and team effectiveness over time will be explained by an increase in group cohesion.

*Hypothesis 3*: Participants who took part in SPT or CBT (vs. no skill training) will display higher levels of coping flexibility over time.

*Hypothesis 4*: The positive association between skill training (vs. no skill training) and coping flexibility over time will be accounted for by an increase in discriminative thinking.

In summary, this study contributed to the literature by adopting an integrative approach to the design of SPT, a novel gamification-based intervention. Instead of focusing on personal skill development *per se* as in the widely adopted CBT, SPT comprises an array of modules designed for strengthening both personal skills (i.e., discriminative thinking) and interpersonal skills (i.e., collective decision-making and social resource accrual) over time. A longitudinal research design was adopted to test the effectiveness of the newly developed SPT. More importantly, a randomized controlled trial was conducted to compare the hypothesized benefits of SPT with two control conditions: existing training control (CBT) and no training control (waitlist). Compared with the participants who were assigned to the waitlist control condition, the participants who took part in the SPT were predicted to experience desirable changes in both team effectiveness and coping flexibility, whereas those who took part in the CBT were predicted to experience desirable changes in coping flexibility only.

## Materials and methods

### Research design

The present study took the form of a randomized controlled trial that adhered to the Consolidated Standards of Reporting Trials (CONSORT). The aim of the trial was to compare the learning mechanisms and outcomes of the newly developed SPT program versus a current CBT program among working adults. Randomized controlled trials are widely regarded as the gold standard for program evaluation (38).

Program effectiveness was assessed using a longitudinal design comprising three time points. Before training began (Time 1/T1), all participants completed the baseline questionnaires at their own work sites after being given instructions by a trained research assistant. Immediately (Time 2/T2) and three months (Time 3/T3) after the training, they completed the follow-up questionnaires on their own.

### Sampling procedures

Employees of our organizational partners in southern China were recruited through a standardized advertisement distributed by the human resource or administration department of each private organization. These departments then submitted lists of the teams who had expressed an interest in taking part.

Eligible participants were full-time employees aged between 18- and 40-years-old who had served in the company for at least six months and could read and communicate in Chinese. Participants were excluded if they reported any psychological illness or medical problems or if they were unwilling to give informed consent or follow the study procedures.

After eligibility screening, an independent research assistant created an allocation schedule using the “random sample of cases” function in SPSS version 26.0 (39), (RRID:SCR_002865). When using this function, the research assistant first entered into the software program the number of teams planned for each of the conditions, and then the software program randomly selected the specified number of teams and assigned them to a skill training program (SPT or CBT) or waitlist.

The study was conducted in accordance with the ethical standards outlined by the American Psychological Association. The research was conducted after obtaining institutional review board approval from the authors’ university. The participants were assured that their participation was entirely voluntary and that their data and performance during the training sessions would be kept strictly confidential and would not be communicated to their employers. Participants assigned to the waitlist control group were told they would be invited to attend SPT sessions if the findings demonstrated the training’s effectiveness. Participants in all three conditions received a souvenir (a pen or towel) for returning their questionnaires at T2 and T3.

### Sample size and statistical power

*A priori* power analysis was performed using G*Power version 3.1 (40), (RRID:SCR_013726) based on the estimated effect size (0.21) obtained in our pilot study [citation redacted for masked review]. The results revealed that a minimum sample size of 54 per condition was sufficient to attain statistical power of at least 90% and to detect significant group differences at a significance level of α = 0.05. Considering the possible attrition rate for a three-phase longitudinal design (41), the target sample size was set at 80 participants per training condition.

### Participants

**FIGURE 1:**
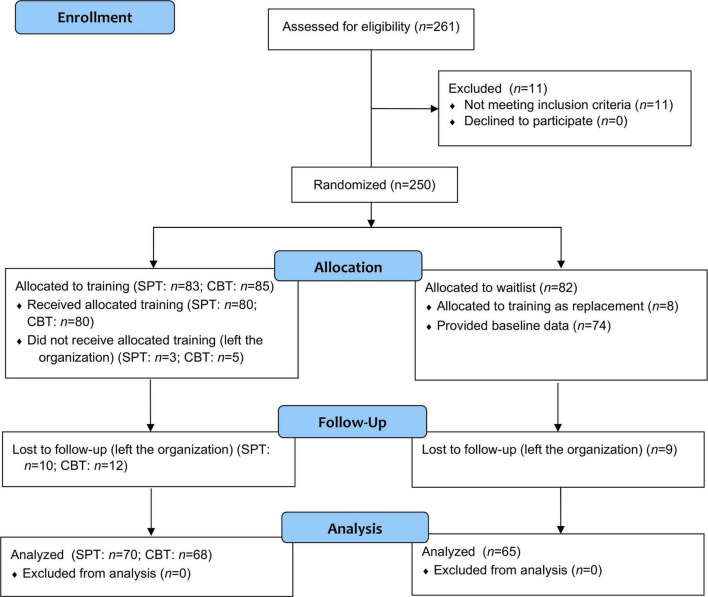
CONSORT diagram of participant flow through various stages.

A total of 234 Chinese working adults were enrolled, with 160 receiving SBT or CBT, but 10, 12, and 9 subsequently dropped out of the SPT, CBT, and waitlist conditions, respectively. Figure 1 depicts the CONSORT flow diagram that summarizes the enrollment and allocation processes. The sample consisted of 68% men, with a median age of 25 years (age range: 18–40). The demographic characteristics of the sample are shown in Table 2. The participants who dropped out and those who took part in all three time points did not differ in any demographic characteristics, *p*s > 0.17.

**TABLE 2:** Descriptive statistics of demographic and study variables by condition.

	Condition					
	SPT (*n* = 70)		CBT (*n* = 68)		Waitlist (*n* = 65)	
	*M* (%)	*SD*	*M* (%)	*SD*	*M* (%)	*SD*
Gender (% of men)	67%		65%		71%	
**Education level**						
Junior secondary education or below	13%		9%		11%	
Senior secondary/vocational education	81%		84%		83%	
Tertiary/university education	6%		7%		6%	
Age	24.93_a_	5.47	25.16_a_	4.51	25.18_a_	4.86
Months of service in the organization	11.58_a_	5.75	11.22_a_	4.66	11.47_a_	5.66
Years of employment	8.22_a_	6.08	7.74_a_	5.02	7.84_a_	5.22
**Team effectiveness**						
T1 self-ratings	15.80_a_	3.88	15.74_a_	3.12	15.52_a_	3.76
T2 self-ratings	17.81_b_	3.04	16.37_a_	3.06	15.75_a_	3.36
T3 self-ratings	18.19_b_	3.56	15.40_a_	2.83	15.28_a_	3.60
T2 actual task performance†	59.34_a_	19.48	63.53_a_	20.32	n/a	
T2 behavioral coding	61.50_b_	19.63	48.37_a_	17.64	n/a	
T3 supervisor-ratings	18.87_b_	2.63	16.63_a_	2.49	16.40_a_	2.83
T1 Coping flexibility	2.68_a_	0.55	2.66_a_	0.54	2.52_a_	0.47
T2 Coping flexibility	2.87_b_	0.48	2.92_b_	0.54	2.62_a_	0.46
T3 Coping flexibility	2.99_b_	0.49	2.83_b_	0.50	2.55_a_	0.48
T1 Group cohesion	8.07_a_	2.68	8.19_a_	2.66	8.15_a_	2.56
T2 Group cohesion	9.83_b_	2.69	8.12_a_	2.73	8.54_a_	2.63
T3 Group cohesion	9.20_b_	2.48	8.19_a_	2.38	8.35_a_	2.48
T1 Discriminative thinking	3.86_a_	1.44	3.66_a_	1.59	3.63_a_	1.66
T2 Discriminative thinking	5.71_c_	1.63	4.75_b_	1.93	3.68_a_	1.76
T3 Discriminative thinking	5.37_b_	1.50	4.99_b_	1.85	3.49_a_	1.63

CBT = cognitive-behavioral training; n/a = not available; SPT = serious play training, T1 = Time 1; T2 = Time 2; T3 = Time 3. †Higher scores indicate greater ineffectiveness in team performance. Means that do not share the same subscripts differ from each other at *p* < 0.05.

### Intervention fidelity

Multiple practices and strategies were undertaken for monitoring and enhancing intervention fidelity in this study. Specifically, the implementation of both SPT and CBT was standardized through the adoption of a manual. Each intervention program had its own manual, which contained comprehensive instructions and thorough descriptions of the content (i.e., goals and objectives, timeframe, scripted text, planned activities, and worksheets) for each of the program sessions.

All the intervention sessions were delivered by an experienced facilitator who was a holder of certificates in both Play Therapy and the LEGO SERIOUS PLAY method. Before the study began, the facilitator received extensive training on the delivery of protocolized procedures for implementing each training program. The facilitator was instructed to closely follow a series of steps outlined in the respective manual of each program.

Implementation fidelity was monitored by two observers, each of whom made the assessment using a checklist adapted from the Implementation Fidelity Checklist constructed by Swain, Finney (42). Each observer gave independent ratings to five categories: program differentiation (SPT vs. CBT), adherence, exposure (planned vs. actual time), quality, and engagement. The inter-observer reliability in the assessment was high across the categories (77% to 97%).

### Skill training programs

Two skill training programs—SPT and CBT—were delivered and compared. Each program involved six bi-weekly two-hour sessions. The two programs were conducted at the organizations from which the participants had been recruited. The newly developed SPT was first piloted on 30 working adults, with the pilot data demonstrating the program’s implementation feasibility and acceptability among participants [citation redacted for masked review].

In both the gamification and instruction conditions, the sessions were delivered to teams of six to eight members. The overarching goal in both conditions was to develop skills that facilitate flexible deployment of coping strategies across the changing environment. The first three modules were psychoeducational, focusing on helping participants to understand the sources of their stress and their own distinct coping styles and then strengthening their discriminative thinking skills to increase coping effectiveness (43).

The three remaining sessions focused on real-life applications, although the delivery mode and context of training differed for the two conditions. A specific feature of the SPT condition was that the modules were delivered to real-life work teams via group games, which combined interactive learning with the LEGO^®^ SERIOUS PLAY^®^ method (44). Active participation in these group games allowed the participants to acquire knowledge and communicate with their real-life team members effectively through immersion in an enjoyable, playful environment (17). Each group game involved three learning phases: LEGO^®^ model building, storytelling, and reflection for learning. Specifically, within a group session, each team was first given a series of stressful vignettes (e.g., conflict with colleagues, reporting a work problem to the supervisor) constructed based on their life stories. In the model building phase, members were instructed to express their solutions through the use of LEGO^®^ bricks to create a model as representations of metaphors. In the storytelling phase, the team conveyed their ideas through presenting their LEGO model and stories to the entire group. In the reflection phase, the entire group discussed their ideas and gave feedback to the presenting team to help the team reflected on their own work. These activities were designed to strengthen the participants’ skills for effective group decision-making and social resource accrual. Through such game engagement, greater cohesion among team members was expected to equip them to deal with work stress effectively.

For the CBT condition, the facilitator provided real-case demonstrations to show the participants how to deploy flexible coping to handle stressful life changes in the same series of vignettes. These activities were designed to strengthen the participants’ skills for effective decision-making and the development of both problem-focused and emotion-focused coping skills (i.e., behavioral activation and relaxation). The content of these modules was designed according to the cognitive-behavioral approach (45). In the final session of both conditions, all the participants were asked to devise their own action plan for future stress management at work and in their daily life.

### Measures

A battery of standardized questionnaires was administered to assess the learning mechanisms and outcome parameters for program evaluation. Specifically, the primary outcome was team effectiveness, whereas the secondary outcomes were coping flexibility. Two learning mechanisms—group cohesion and discriminative thinking—were also assessed.

For all these outcomes and learning mechanisms, self-ratings were obtained from the participants in all three conditions at all three time points. In addition, the primary outcome of team effectiveness was thoroughly evaluated using four methods: self-ratings, supervisor-ratings, actual task performance, and observation (behavioral coding). Data derived from actual task performance and observation were collected during discussions held in the final training session for both the SPT and CBT conditions. Supervisor-ratings were collected for the participants after the training at T3.

#### Team effectiveness

Both the self- and supervisor-ratings of team effectiveness were measured using the Behaviorally Anchored Rating Scale version of the Comprehensive Assessment of Team Member Effectiveness measure (46). Each of the five domains of team effectiveness was evaluated by a 5-point rating, with each rating point anchored by a set of behavioral indicators. The raters were instructed to read all the behavioral indicators and then choose the response option that best reflected the actual team behaviors. The composite scores ranged from 5 to 25. The translated measure has been validated for the assessment of both self- and other-ratings in Chinese samples (47).

Team (vs. individual) performance during the training sessions was evaluated by a decision-making task entitled “Winter survival” (48). The task presented a stressful vignette regarding a group of people who survived from a plane crash but encountered a dire situation in a wilderness area under a severely cold weather. The group managed to salvage 12 items when escaped from the plane, and the task was to rank the set of items to indicate the importance of each item to their survival in the dire situation. Team members first gave their own sets of rankings independently, and then engaged in discussion to generate final team decisions. Team performance was computed by the absolute sum differences between a team’s group rankings and the expert rankings. As lower difference scores indicated greater accuracy, a higher discrepancy score indicated less effective team performance. This task was administered in the final session of the SPT and CBT conditions at T2.

Each team member’s effective (vs. ineffective) teamwork behavior during the decision-making process at T2 was recorded and coded by two independent observers. These extensively trained observers coded the team behaviors exhibited by all team members while they engaged in the “Winter survival” task using the Behavioral Observation Scale (49). The observers adopted a 7-point scale (1 = almost never, 7 = almost always) to rate 16 teamwork categories. The ratings for two ineffective behavioral markers were reverse scored, with higher composite scores indicating a team member’s greater display of effective teamwork behavior. The level of inter-observer reliability was high (Krippendorff’s α ≥ 0.70).

#### Coping flexibility

The Coping Flexibility Questionnaire (50) was adopted as an indicator of flexible coping. The inventory comprised two sections. In the first section, respondents were asked to list two controllable and two uncontrollable stressful events. In the second section, they reported up to four coping strategies and their corresponding goals for coping with each event. The respondents’ coping goals were scored according to a validated scoring scheme based on the transactional theory of coping (51). The final codings ranged from 0 to 4, with higher scores indicating greater coping flexibility. This coping measure is found reliable and valid in Chinese samples (50).

#### Group cohesion

The Group Cohesion Scale (52) was employed to assess group cohesion perceptions. Respondents rated each of the scale’s three items on a Likert scale ranging from 1 (strongly disagree) to 5 (strongly agree). Higher scores indicated stronger perceptions of cohesion among team members. The composite scores ranged from 3 to 15. The translated measure has been validated in Chinese settings (53).

#### Discriminative thinking

The Extended Miller Behavioral Style Scale (37) was used as a measure of discriminative thinking. The brief version of the scale comprises two hypothetical stressful vignettes, each with eight coping responses. Respondents were instructed to endorse the deployment of each coping response in each vignette (0 = no, 1 = yes). The items were scored by a scheme derived by experts (37). The final codings ranged from 0 to 16, with higher values indicating a greater tendency toward discriminative thinking. This scale has good psychometric properties in Chinese samples (37).

### Manipulation checks

The participants were asked to guess the purpose of the study and to report whether they had previously seen any decision-making tasks similar to that used in the present study. None of the participants could provide a correct guess of the study aims, and none had seen this type of task before.

### Masking

Participants’ personal data and the condition to which they were assigned were concealed by arbitrary codes. The randomized controlled trial involved single masking, because the facilitator needed to administer different intervention programs to participants of various skill training conditions. Nevertheless, partial masking was adopted for different research teams to maximize the level of masking. Specifically, the supervisors who gave ratings, the observers who conducted behavioral codings, and the research assistants who administered the study and input data were unaware of the allocation schedule, randomization procedures, and research hypotheses. Moreover, the investigators were unaware of the allocation schedule and randomization procedures during their interpretation of the findings.

### Analytical strategy

Before hypothesis testing, outlier analysis was performed. Multiple outlier identification methods—boxplot, quantile-quantile plot, and stem-and-leaf plot—were employed to detect as many outliers as possible. Outliers identified by the three methods were removed. If the results obtained after outlier removal differed from those derived from the full sample, both sets of results are reported herein. If no substantial differences in the pattern of findings were found, the full results would be reported.

For outcome evaluations, the hypothetical longitudinal changes in the primary (team effectiveness) and secondary (coping flexibility) outcomes among the participants of the three conditions were tested by linear mixed effects modeling using analysis of covariance with three fixed factors (condition, time, and condition by time) and the baseline value as covariate. The missing data for the analysis of covariance modeling neither were imputed nor were they carried forward.

To unveil the hypothetical mechanisms underlying outcome changes, mediation analysis was performed using PROCESS macro version 3.5 (54), (RRID:SCR_021369). Model 4 with the widely adopted, standard procedure of 5,000 bootstrap simulations was executed (55). In each mediation model, training condition (SPT, CBT, and waitlist) was entered as the antecedent, the hypothesized mechanisms (group cohesion and discriminative thinking) assessed at T2 were the mediator, and the primary and secondary outcomes assessed at T3 were included as the outcome. The baseline levels of both the mediator and outcome variables were controlled. The significance of indirect effects was assessed by 95% bias-corrected and accelerated bootstrap (BCa) confidence intervals (CIs). To test the antecedent of training condition, the three-level categorical variable was coded into two dummy variables: SPT (1 = SPT, 0 = CBT/waitlist) and skill training (1 = SPT/CBT, 0 = waitlist). All analyzes were conducted using SPSS 26.0 (39), (RRID:SCR_002865).

## Results

### Preliminary analyzes

Outliers detected by all three outlier identification methods were checked, and none were found to be error outliers. To further identify influential outliers, all outliers were omitted, with the same set of main analyzes conducted again. The pattern and interpretation of the findings yielded from the full sample and the trimmed sample with all outliers removed did not differ substantially, indicating that none of the suspected cases was an influential outlier.

Table 2 presents the descriptive statistics of both the demographic and study variables for the three conditions. No significant differences were found for any of the demographic variables (*p*s > 0.09), with the exception of a significant positive association between length of service in the organization and self-ratings of team effectiveness reported at T1, *r*(203) = 0.14, *p* = 0.04. The analyzes were thus conducted with the pooled sample, with no demographic variables included as covariates.

### Evaluations of program effectiveness

The efficacy of SPT (vs. CBT and waitlist conditions) was evaluated in terms of changes in both team effectiveness and coping flexibility over time. For the participants’ self-ratings of team effectiveness, the hypothesized Condition × Time interaction was significant, *F*(4, 600) = 3.75, *p* = 0.005, Cohen’s *f* = 0.13. The participants of the three conditions did not differ in their ratings of team effectiveness at T1, but those assigned to the SPT condition gave higher team effectiveness ratings at T2 and T3 than those assigned to the other two conditions (*p*s < 0.02).

For the supervisors’ ratings of team effectiveness at T3, significant differences among the three conditions were also found, *F*(2, 200) = 18.19, *p* < 0.001, Cohen’s *f* = 0.41. The supervisors gave higher ratings of team effectiveness for the participants in the SPT condition than for those in the two other conditions.

For the observer-coding of actual team behavior at T2, the observers gave higher scores for the effective team behaviors of the participants taking part in SPT (vs. CBT), *t*(136) = 4.14, *p* < 0.001, Hedges’ *g* = 0.70; but no such differences in task performance scores were found between the two conditions, *t*(136) = –1.24, *p* = 0.22, Hedges’ *g* = –0.21. Taken together, all these findings derived from multiple methods were largely consistent with Hypothesis 1.

The efficacy of both skill training programs (i.e., both SPT and CBT) was assessed in terms of changes in coping flexibility over time. A linear mixed effect model revealed that the hypothesized Condition × Time interaction was not significant, *F*(4, 600) = 1.60, *p* = 0.17, Cohen’s *f* = 0.06. However, significant main effects were found for condition, *F*(2, 600) = 18.31, *p* < 0.001, Cohen’s *f* = 0.24; and for time, *F*(2, 600) = 8.46, *p* < 0.001, Cohen’s *f* = 0.16. There were no differences among the participants of the three conditions at T1, but those assigned to the SPT and CBT conditions exhibited greater coping flexibility at T2 and T3 than those assigned to the CBT or waitlist conditions (*p*s < 0.001). Such findings provided partial support for Hypothesis 2.

### Mechanisms for explaining program effectiveness

**FIGURE 2:**
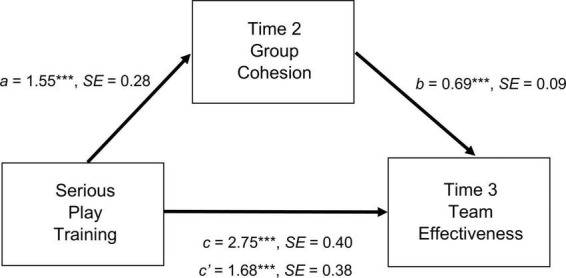
Indirect effects of serious play training on team effectiveness assessed at time 3 through group cohesion assessed at time 2. Mediation analysis was performed using PROCESS (model 4; Hayes, (55)) with 5,000 bootstrapping resamples. Baseline levels of group cohesion and team effectiveness were entered as covariates. Serious play training was dummy coded (1 = serious play training, 0 = cognitive-behavioral training and waitlist). a, b, c, and c’ are unstandardized coefficients. ***p < 0.001.

**FIGURE 3:**
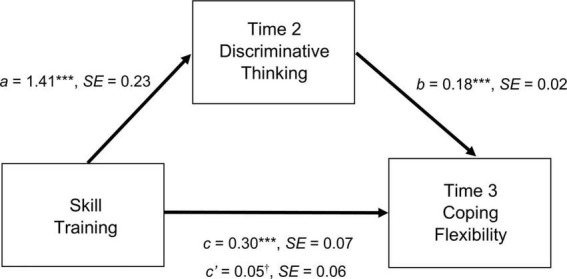
Indirect effects of skill training on coping flexibility assessed at time 3 through discriminative thinking assessed at time 2. Mediation analysis was performed using PROCESS (Model 4; Hayes, (55)) with 5,000 bootstrapping resamples. Baseline levels of discriminative thinking and coping flexibility were entered as covariates. Skill training was dummy coded (1 = serious play training and cognitive-behavioral training, 0 = waitlist). a, b, c, and c’ are unstandardized coefficients. ***p < 0.001; ^†^*p* > 0.05.

To test the hypothesized mechanisms underlying the beneficial changes in the acquisition of flexible coping skills after attending SPT, mediation analysis was conducted. The results are summarized in Figures 2 and 3. Referring to Figure 2, the hypothesized indirect effect of SPT (vs. CBT and waitlist) on T3 self-ratings of team effectiveness through T2 group cohesion was significant, but no such mediation effects were found for T2 discriminative thinking. The direct effect remained significant after controlling for the effect of T2 group cohesion, indicating the presence of a partial mediation. The results thus supported Hypothesis 3.

Finally, the hypothesized mechanisms underlying the beneficial changes in coping flexibility after attending SPT or CBT were also tested. The findings are shown in Figure 3. Referring to this figure, the hypothesized indirect effect of skill training on T3 coping flexibility through T2 discriminative thinking was significant. The direct effect (path c’) became non-significant after controlling for the effect of the mediator, thus indicating complete mediation. These findings provided support for Hypothesis 4.

## Discussion

This study introduced a newly designed SPT program as an alternative approach to staff development that strengthens Chinese workers’ personal and interpersonal skills, both of which are beneficial to team building and flexible coping with life stress. Through examining the learning mechanisms underlying program participation, the findings demonstrate that SPT has the advantage over CBT in strengthening team effectiveness, with such a desirable change explained by an increase in group cohesion. In addition, the findings further provide evidence suggesting that SPT is as effective as existing CBT programs in enhancing coping flexibility over time, with the strengthening of discriminative thinking skills found to explain this positive change.

For team building, only SPT is found to enhance team effectiveness. Such encouraging findings provide support for the use of serious play as an adult learning tool in team building, one of the most popular employee development themes in corporate training. The serious play method is grounded in theories of play, imagination, and complexity; aiming to uncover and create new insights by using toy materials (e.g., LEGO bricks, doll house) as a medium for visualizing, communicating, understanding, and tackling challenges (56). The major advantage is the method’s playful, hands-on approach, which turns a work setting into a constructive playground. The fun and relaxing environment gently pushes team members to think “outside the box,” facilitating them to brainstorm more unusual ideas and speak their minds effectively to an audience (57).

The findings also support the hypothesized mediating role of group cohesion in the link between SPT participation and self-rated team effectiveness. However, it is noteworthy that this mediation effect is only partial. This result may be attributable to the highly complex nature of team effectiveness, which is a multifaceted construct (58). Previous work has indicated that team effectiveness comprises a variety of domains, including both task-oriented (e.g., leadership, coordination) and social-oriented (e.g., communication, conflict management) domains (59). Future program evaluation studies should adopt a more nuanced approach to identify the specific domains and psychological mechanisms that account for greater variances in outcome changes resulting from SPT participation. Nevertheless, nuanced approaches can have shortcomings because many learning processes involve the simultaneous occurrence of multiple psychological mechanisms that are practically difficult to dissect for separate analyzes.

For stress management, the newly introduced SPT approach is found to be as effective as the existing, popular method of CBT in fostering coping flexibility, demonstrating that SPT is an effective tool for building not only team but also personal skills. In corporate training, the serious play intervention has been adopted for change management so as to improve the company and promote its future development. It is important to note that the aim of the intervention is not just to foster creativity and flexibility among company staff, but ultimately to help transform the staff and their company (60). The creative and flexible thinking skills acquired from SPT enable the participants not only to spot new opportunities in the business environment, but more importantly, to change strategies, structures, products, business models, and systems. In the same vein, our study further shows that the SPT is also efficacious for facilitating coping flexibility, an essential skill for handling stressful life transitions and vicissitudes (5). Workshop participants acquire cognitive astuteness and sensitivity to environmental cues (i.e., cognitive flexibility) through constant shifting of their constructed models from one context to another, and their acquired astuteness facilitates the choice of the most situation-appropriate strategy from their coping repertoire (i.e., behavioral flexibility) (61).

Our study further unveils discriminative thinking as a cognitive mechanism underlying the building of flexible coping skills. In SPT sessions, toy materials (i.e., LEGO bricks) are used as a major tool for supporting flexible thinking, developing a creative culture, and contributing to learning processes through storytelling and reflection (17). The bricks are flexible for building symbolic and metaphorical models. When completing a single task in a training session, participants can easily build and rebuild their models by removing the bricks and placing the bricks in other locations. They are also asked to switch contexts by creating new stories and building new models. Such frequent task switching enables the participants to acquire discriminative thinking, which fosters deployment of a coping strategy that best meets the demands of a particular stressful encounter (62, 63).

The novel findings presented herein have practical implications for corporate training with a special focus on personal development. Most existing intervention programs in this area adopt an individual-oriented approach aimed at building cognitive-behavioral skills that can facilitate the effective handling of stressful events (64). This widely adopted approach has been criticized for largely neglecting a crucial dimension of the occupational environment, such as the organizational and interpersonal elements at play (11).

To address this important but unexplored dimension, we recommend a paradigmatic shift toward an integrative, ecologically relevant approach that broadens the scope of skill development to both personal and interpersonal realms in real-life work teams. The aim of this alternative novel approach is to attain the goal of skill acquisition through real-life social engagement. Our proposed SPT expands upon the often-demarcated designs that are targeted primarily at the individual level, thereby acknowledging the need to concurrently attend to workshop participants’ interactions with their actual surroundings and immediate contextual characteristics (e.g., peer relations, institutional demands, cultural orientation), which constitute ever-evolving psychosocial stressors inherent to the work environment.

The major advantage of this novel approach is that SPT training activities are designed based on the real-life work problems reported by employees with the same work and cultural backgrounds, and thus are of direct relevance to the participants’ interests that increase their motivation for active participation and engagement. Despite the workshop setting, participants have already been practicing their acquired skills through interacting with members of their real-life work teams. Under the guidance of a qualified facilitator, participants can further polish their coping skills through reflection on their dynamic exchanges during the sessions and collaborations with their fellow team members in serious games, as well as meaning-making through social interactions, thereby facilitating the active construction and consolidation of knowledge acquired in the training sessions (14, 15).

Although the present study contributes to the literature by providing evidence supporting the efficacy of a new intervention approach to corporate training, several limitations suggest that broader generalizations of the findings should be made with caution. First, SPT in this study focuses solely on the cultivation of coping skills. The gamification approach may not be equally effective for the acquisition of other personal development skills such as leadership and assertiveness. The scope of SPT should be expanded to an array of skills to allow a more thorough evaluation of its usefulness in corporate training.

Moreover, as the study targeted the training of actual teams within real organizations, participant recruitment was arranged by the administrative staff of the participating organizations. The sample was thus relatively homogeneous, constituting mainly young men who had attained a senior secondary level of education or received vocational education. Hence, the present findings may not be generalizable to employees with dissimilar demographic characteristics.

## Conclusion

The present study is the first to test the efficacy of the newly introduced SPT intervention approach and compare it with the existing CBT approach in terms of both team and personal skill training. As expected, only SPT is found to strengthen team building skills over time, and group cohesion is identified as the underlying learning mechanism that explains this positive change. In addition, SPT is found to be as beneficial as the existing CBT in sharpening flexible coping skills, and discriminative thinking is identified as the learning mechanism underlying the positive change. Such promising results encourage future researchers and practitioners to draw more attention and resources to utilize SPT for developing a broader array of skills in both corporate and public wellness promotion programs.

## Data availability statement

The raw data supporting the conclusions of this article will be made available by the authors, without undue reservation.

## Ethics statement

The studies involving human participants were reviewed and approved by Human Research Ethics Committee (HREC) of the University of Hong Kong. The patients/participants provided their written informed consent to participate in this study.

## Author contributions

CC contributed to the conception and design of the study, coordinated the data collection process, performed the statistical analysis, and wrote the first draft of the manuscript. C-LC wrote sections of the manuscript. Both authors conducted the literature review, contributed to the editing and revision of the manuscript, and approved the submitted version.

## References

[1] Frese M. The changing nature of work. 3rd ed. In: Chmiel N Fraccaroli F Sverke M editors. *An Introduction to Work and Organizational Psychology.* (Hoboken, NJ: Blackwell) (2017). p. 397–413.

[2] Johnson JV Lipscomb J. Long working hours, occupational health and the changing nature of work organization. *Am J Ind Med.* (2006) 49:921–9. 10.1002/ajim.20383 16986150

[3] Cheng C Wang H-Y Ebrahimi OV. Adjustment to a “new normal”: coping flexi(bility and mental health issues during the COVID-19 pandemic. *Front Psychiatry.* (2021) 12:626197. 10.3389/fpsyt.2021.626197 33815166 PMC8017149

[4] Mendonça I Coelho F Ferrajão P Abreu AM. Telework and mental health during COVID-19. *Int J Environ Res Public Health.* (2022) 19:2602. 10.3390/ijerph19052602 35270294 PMC8909524

[5] Cheng C Lau HB Chan MS. Coping flexibility and psychological adjustment to stressful life changes: a meta-analytic review. *Psychol Bull.* (2014) 140:1582–607. 10.1037/a0037913 25222637

[6] Giga SI Noblet AJ Faragher B Cooper CL. The UK perspective: a review of research on organisational stress management interventions. *Aust Psychol.* (2003) 38:158–64. 10.1080/00050060310001707167

[7] Richardson KM Rothstein HR. Effects of occupational stress management intervention programs: a meta-analysis. *J Occup Health Psychol.* (2008) 13:69. 10.1037/1076-8998.13.1.69 18211170

[8] Fjorback LO Arendt M Ørnbøl E Fink P Walach H. Mindfulness-based stress reduction and mindfulness-based cognitive therapy–a systematic review of randomized controlled trials. *Acta Psychiatr Scand.* (2011) 124:102–19. 10.1111/j.1600-0447.2011.01704.x 21534932

[9] Hundt NE Mignogna J Underhill C Cully JA. The relationship between use of CBT skills and depression treatment outcome: a theoretical and methodological review of the literature. *Behav Ther.* (2013) 44:12–26. 10.1016/j.beth.2012.10.001 23312423

[10] Zion M Mendelovici R. Moving from structured to open inquiry: challenges and limits. *Sci Educ Int.* (2012) 23:383–99.

[11] Tetrick LE Winslow CJ. Workplace stress management interventions and health promotion. *Annu Rev Organ Psychol Organ Behav.* (2015) 2:583–603. 10.1146/annurev-orgpsych-032414-111341

[12] Pitsoe VJ. From an instructionist to a constructivist classroom management: a dialogue. *Int J Educ Sci.* (2014) 7:391–9. 10.1080/09751122.2014.11890245

[13] Spiro RJ Collins BP Thota JJ Feltovich PJ. Cognitive flexibility theory: hypermedia for complex learning, adaptive knowledge application, and experience acceleration. *Educ Technol.* (2003) 43:5–10.

[14] Amineh RJ Asl HD. Review of constructivism and social constructivism. *J Soc Sci.* (2015) 1:9–16.

[15] Hendry GD Frommer M Walker RA. Constructivism and problem-based learning. *J Furth High Educ.* (1999) 23:369–71. 10.1080/0309877990230306

[16] Hofstede GH. *Culture’s Consequences: Comparing Values, Behaviors, Institutions, and Organizations Across Nations.* 2nd ed. Thousand Oaks, CA: Sage (2001).

[17] Gauntlett D. The LEGO System as a tool for thinking, creativity, and changing the world. In: Gauntlett D editor. *Making Media Studies: The Creativity Turn in Media and Communications Studies.* (New York, NY: Peter Lang) (2015). p. 1–15.

[18] Dicheva D Dichev C Agre G Angelova G. Gamification in education: a systematic mapping study. *J Educ Techno Soc.* (2015) 18:75–88. 10.1016/j.heliyon.2019.e01993 31360779 PMC6639688

[19] Hoffmann A Christmann CA Bleser G. Gamification in stress management apps: a critical app review. *JMIR Serious Games.* (2017) 5:e7216. 10.2196/games.7216 28592397 PMC5480012

[20] AlMarshedi A Wanick V Wills GB Ranchhod A. Gamification and behaviour. In: Stieglitz S Lattemann C Robra-Bissantz S Zarnekow R Brockmann T editors. *Gamification.* (Berlin: Springer) (2017). p. 19–29. 10.1007/978-3-319-45557-0_2

[21] Prasad JRV Alexander J Misra S. Gamification and employees’ perception: an empirical evaluation using gamification effectiveness Scale. *Int J Manag Bus Res.* (2019) 9:19–27.

[22] Johnson D Deterding S Kuhn K-A Staneva A Stoyanov S Hides L. Gamification for health and wellbeing: a systematic review of the literature. *Internet Interv.* (2016) 6:89–106. 10.1016/j.invent.2016.10.002 30135818 PMC6096297

[23] Lowensteyn I Berberian V Berger C Da Costa D Joseph L Grover SA. The sustainability of a workplace wellness program that incorporates gamification principles: participant engagement and health benefits after 2 years. *Am J Health Promot.* (2019) 33:850–8. 10.1177/0890117118823165 30665309

[24] Chen S Fan Y Zhang G Zhang Y. Collectivism-oriented human resource management on team creativity: effects of interpersonal harmony and human resource management strength. *Int J Hum Resour.* (2019) 6:1–28.

[25] Chen S Zhang G Zhang A Xu J. Collectivism-oriented human resource management and innovation performance: an examination of team reflexivity and team psychological safety. *J Manag Organ.* (2016) 22:535. 10.1017/jmo.2015.50

[26] Luu S Narayan A. Games at work: examining a model of team effectiveness in an interdependent gaming task. *Comput Hum Behav.* (2017) 77:110–20. 10.1016/j.chb.2017.08.025

[27] Uz Bilgin C Gul A. Investigating the effectiveness of gamification on group cohesion, attitude, and academic achievement in collaborative learning environments. *TechTrends.* (2020) 64:124–36. 10.1007/s11528-019-00442-x

[28] Andreassi JK Lawter L Brockerhoff M Rutigliano PJ. Cultural impact of human resource practices on job satisfaction. *Cross Cult Manag.* (2014) 21:55–77. 10.1108/CCM-05-2012-0044

[29] Reinig BA Shin B. The dynamic effects of group support systems on group meetings. *J Manag Inform Syst.* (2002) 19:303–25. 10.1080/07421222.2002.11045728

[30] Scott RJ Cavana RY Cameron D. Recent evidence on the effectiveness of group model building. *Eur J Oper Res.* (2016) 249:908–18. 10.1016/j.ejor.2015.06.078

[31] Cheng C. Cognitive and motivational processes underlying coping flexibility: a dual-process model. *J Pers Soc Psychol.* (2003) 84:425–38. 10.1037/0022-3514.84.2.425 12585814

[32] Chiu C Hong Y Mischel W Shoda Y. Discriminative facility in social competence: conditional versus dispositional encoding and monitoring-blunting of information. *Soc Cogn.* (1995) 13:49–70. 10.1521/soco.1995.13.1.49

[33] Mischel W Shoda Y. A cognitive-affective system theory of personality: reconceptualizing situations, dispositions, dynamics, and invariance in personality structure. In: Higgins ET Kruglanski AW editors. *Motivational Science: Social and Personality Perspectives.* (Philadelphia, PA: Psychology Press) (2000). p. 150–76. 10.1037/0033-295x.102.2.246

[34] Ellwood S Pallier G Snyder A Gallate J. The incubation effect: hatching a solution? *Creat Res J.* (2009) 21:6–14. 10.1080/10400410802633368

[35] Koppel RH Storm BC. Escaping mental fixation: incubation and inhibition in creative problem solving. *Memory.* (2014) 22:340–8. 10.1080/09658211.2013.789914 23607286

[36] Lu J Lajoie SP Wiseman J. Scaffolding problem-based learning with CSCL tools. *Int J Comput Support Collab Learn.* (2010) 5:283–98. 10.1007/s11412-010-9092-6

[37] Cheng C Chiu C Hong Y Cheung JS. Discriminative facility and its role in the perceived quality of interactional experiences. *J Pers.* (2001) 69:765–86. 10.1111/1467-6494.695163 11575513

[38] McGovern DPB. Randomized controlled trials. In: McGovern DPB Valori RM Summerskill WSM editors. *Key Topics in Evidence-Based Medicine.* (Oxford: BIOS Scientific) (2001). p. 26–9.

[39] IBM Corp. *IBM SPSS Statistics for Windows, Version 26.0.* Armonk, NY: IBM Corp (2019).

[40] Faul F Erdfelder E Lang A Buchner A. G*Power 3: a flexible statistical power analysis program for the social, behavioral, and biomedical sciences. *Behav Res Methods.* (2007) 39:175–91. 10.3758/bf03193146 17695343

[41] Sandberg M Kristensson J Midlöv P Jakobsson U. Effects on healthcare utilization of case management for frail older people: a randomized controlled trial (RCT). *Arch Gerontol Geriatr.* (2015) 60:71–81. 10.1016/j.archger.2014.10.009 25459920

[42] Swain MS Finney SJ Gerstner JJ. A practical approach to assessing implementation fidelity. *Assess Update.* (2013) 25:5–7.

[43] Cheng C Kogan A Chio JH. The effectiveness of a new, coping flexibility intervention as compared with a cognitive-behavioural intervention in managing work stress. *Work Stress.* (2012) 26:272–88. 10.1186/s12913-016-1423-5 27409075 PMC4943498

[44] Kristiansen P Rasmussen R. *Building a Better Business Using the Lego Serious Play Method.* Hoboken, NJ: Wiley (2014).

[45] Sheldon B. *Cognitive-Behavioral Therapy: Research, Practice, and Philosophy.* London: Routledge (1995).

[46] Ohland MW Loughry ML Woehr DJ Bullard LG Felder RM Finelli CJ The comprehensive assessment of team member effectiveness: development of a behaviorally anchored rating scale for self- and peer evaluation. *Acad Manag Learn Educ.* (2012) 11:609–30. 10.5465/amle.2010.0177

[47] Lau P Kwong T Chong K Wong E. Developing students’ teamwork skills in a cooperative learning project. *Int J Lesson Learn Stud.* (2013) 3:80–99.

[48] Johnson DW Johnson FP. *Joining Together: Group Theory and Group Skills.* 11th ed. Hoboken, NJ: Prentice Hall (2013).

[49] Taggar S Brown TC. Problem-solving team behaviors: development and validation of BOS and a hierarchical factor structure. *Small Group Res.* (2001) 32:698–726. 10.1177/104649640103200602

[50] Cheng C. Assessing coping flexibility in real-life and laboratory settings: a multimethod approach. *J Pers Soc Psychol.* (2001) 80:814–33. 11374752 10.1037//0022-3514.80.5.814

[51] Lazarus RS Folkman S. Transactional theory and research on emotions and coping. *Eur J Pers.* (1987) 1:141–69. 10.1002/per.2410010304

[52] Podsakoff PM MacKenzie SB. An examination of the psychometric properties and nomological validity of some revised and reduced substitutes for leadership scales. *J Appl Psychol.* (1994) 79:702–13. 10.1037/0021-9010.79.5.702

[53] Wu C Neubert MJ Xiang Y. Transformational leadership, cohesion perceptions, and employee cynicism about organizational change: the mediating role of justice perceptions. *J Appl Behav Sci.* (2007) 43:327–51. 10.1177/0021886307302097

[54] Hayes AF. The PROCESS Macro for SPSS and SAS [Computer Software]. (2019).

[55] Hayes AF. *Introduction to Mediation, Moderation, and Conditional Process Analysis: A Regression-Based Approach.* 2nd ed. New York, NY: Guilford (2018).

[56] Grienitz V Schmidt AM. Scenario workshops for strategic management with Lego serious play. *Probl Manag 21st Century.* (2012) 3:26–36. 10.33225/pmc/12.03.26

[57] Geithner S Menzel D. Effectiveness of learning through experience and reflection in a project management simulation. *Simul Gaming.* (2016) 47:228–56. 10.1177/1046878115624312

[58] Hackman JR. *Groups That Work and Those That Don’t.* San Francisco, CA: Jossey-Bass (1990).

[59] Rowland P Lising D Sinclair L Baker GR. Team dynamics within quality improvement teams: a scoping review. *Int J Qual Health Care.* (2018) 30:416–22. 10.1093/intqhc/mzy045 29617795

[60] Karp T. Unpacking the mysteries of change: mental modelling. *J Change Manag.* (2005) 5:87–96. 10.1080/14697010500057573

[61] Cheng C Chau C. When to approach and when to avoid? Functional flexibility is the key. *Psychol Inq.* (2019) 30:125–9. 10.1080/1047840X.2019.1646040

[62] Cheng C Chan N Chio JH Chan P Chan AO Hui W. Being active or flexible? Role of control coping on quality of life among patients with gastrointestinal cancer. *Psychooncology.* (2012) 21:211–8. 10.1002/pon.1892 22271542

[63] Cheng C Cheung MWL. Cognitive processes underlying coping flexibility: differentiation and integration. *J Pers.* (2005) 73:859–86. 10.1111/j.1467-6494.2005.00331.x 15958137

[64] Kular NK. Stress management: concept. *Employees and Employers in Service Organizations*, ed. Birdie A. K. (New Jersey, NJ: Apple Academic) (2017). p. 291–334. 10.1201/9781315365855-13

